# Recent Advances in the Treatment of Industrial Wastewater from Different Celluloses in Continuous Systems

**DOI:** 10.3390/polym15193996

**Published:** 2023-10-05

**Authors:** Uriel Fernando Carreño Sayago, Vladimir Ballesteros Ballesteros

**Affiliations:** Engineering Faculty, Fundacion Universitaria los Libertadores, Bogotá 111221, Colombia; vladimir.ballesteros@libertadores.edu.co

**Keywords:** crassipes, vegetable, biomass, adsorption, model

## Abstract

There are numerous studies on water care methods featured in various academic and research journals around the world. One research area is cellulose residue coupled with continuous systems to identify which are more efficient and easier to install. Investigations have included mathematical design models that provide methods for developing and commissioning industrial wastewater treatment plants, but nothing is provided on how to size and start these treatment systems. Therefore, the objective is to determine recent advances in the treatment of industrial wastewater from different celluloses in continuous systems. The dynamic behavior of the research results with cellulose biomasses was analyzed with the mass balance model and extra-particle and intraparticle dispersion, evaluating adsorption capacities, design variables, and removal constants, and making a size contribution for each cellulose analyzed using adsorption capacities. A mathematical model was also developed that feeds on cellulose reuse, determining new adsorption capacities and concluding that the implementation of cellulose waste treatment systems has a high feasibility due to low costs and high adsorption capacities. Furthermore, with the design equations, the companies themselves could design their systems for the treatment of water contaminated with heavy metals with cellulose.

## 1. Introduction

The world is experiencing a fearsome reality, which is the increasingly unavoidable scarcity of drinking water, often due to the irresponsibility of companies that do not have adequate treatment for their industrial wastewater [1,2,3]. The dumping of untreated heavy metals into different water bodies leads to a frightening reality due to its irreversible impacts in many cases [4,5,6,7]. Through research projects in the area of water treatment, compliance with the objectives of sustainable development could be achieved, such as clean water and sanitation, and indirectly the rest of these objectives [8,9,10].

A novel approach with reliable results is the development of treatment systems with residual cellulose biomass. This polymer can retain heavy metals due to cation exchange processes via chemisorption [11,12,13]. The unique hierarchical porous structure of cellulose is very conducive to fluid passage while absorbing small particles in the intercepted fluid [14].

One way to improve the removal of heavy metals from cellulose is through their chemical or physical modification to increase their affinity for contaminant molecules [15,16,17].

In both laboratory and pilot scale research, it is being concluded that through continuous systems, treatment systems could be installed to remove heavy metals from water, designing and developing mathematical models to adjust them to the established needs [18,19]. For example, the extra-particle diffusion equation could be adapted to the needs of contaminated wastewater treatment systems. In addition, this equation could be adapted to the needs in processing the final concentration of heavy metals, adapting it to the current discharge legislation [20,21,22,23,24].

Another model worthy of investigation is the intraparticle diffusion model that links microparticle densities and biomass density, relating them to establish the best design conditions [25,26,27,28,29]. To establish these treatment conditions, the design parameters of the system must be known, including density ratios, design flow rate, removal capacity, treatment biomass, breakpoint, and design volume. With this information, equations were developed to establish the initial conditions to achieve the heavy metal release targets [30,31]. Fixed bed column adsorption tests are used to establish the efficiency of biomass in pollutant removal, in a similar arrangement to that used in industry [32,33,34]. To optimize the treatment process, the use of chemical agents such as EDTA, HCl, NaOH, and HNO_3_ is essential to reuse cellulose in the treatment of heavy metals, and therefore increase the adsorption capacity [35,36,37]. Cellulose can assimilate these chemical agents due to the presence of lignin in its biochemical structure, which helps to maintain its stringency in the processing process without being affected by it, allowing up to six times more recycling in heavy metal adsorption processes [38,39,40]. Traditional adsorbents have shortcomings, such as low adsorption capacity and low selectivity, so it is urgent to develop new adsorbents with high adsorption capacity, and which are renewable and without secondary contamination [41]. The adaptation of cellulose in treatment systems could be carried out through a fixed-bed column with PET plastic containers; this guarantees it has an economic advantage, being easy to implement and effective compared to other methods [9].

For this reason, the present review work aims to determine the recent advances in the treatment of industrial wastewater from different celluloses in continuous systems. The dynamic behavior of the results of the biomass research will be analyzed with the mass balance model and extra-particle and intraparticle diffusion, where adsorption capacities, design variables, and removal constants were evaluated and staging and sizing contributions were made for each cellulose analyzed for adsorption capacities. A mathematical model was also developed, where the elution of contaminant removal was fed to determine the new adsorption capacities, and conclude which is the chemical agent with the best performance in the reuse of cellulose. In addition, some system developments with cellulose biomass are shown, and the cost of these systems is analyzed.

## 2. Mechanism of Adsorptions and Characterizations of Cellulose

### 2.1. Analysis FTIR of Cellulose

Different investigations related to cellulose FTIR were analyzed, and the generic figure of heavy metal adsorption in cellulose was obtained.

**Figure 1:**
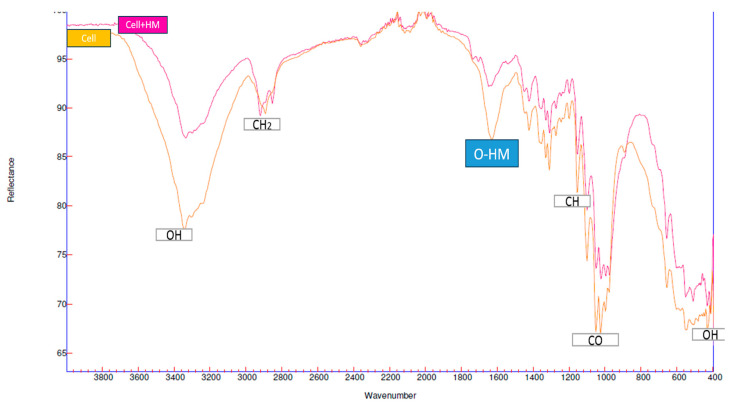
FTIR characterizations of cellulose.

Figure 1 shows the characteristic spectra of a cellulose sample before and after the adsorption of heavy metals; for example, the hydroxyl groups (-OH) can be observed in the bands 3400 cm^−1^ and 580 cm^−1^ [42,43,44,45,46,47]. After the experimental process of adsorption of a heavy metal–light green spectrum, significant changes in its stretching levels were observed, displaying an important change in the hydroxyl group (OH) after the chemisorption process. Parts of the hydroxyl groups are lost in the characterization due to the presence in the cellulose of a heavy metal due to the strong vibrations of the O-HV [9]. FTIR spectra were used to show the binding of Cr (VI) ions in the -OH group, which contributed to the adsorption of this heavy metal in the biomass of *E. crassipes*. Other studies also found that -OH was involved in the adsorption process of different heavy metals, and in these investigations they modified cellulose in order to increase these groups, in order to increase the removal levels of these contaminants [48,49,50,51].

### 2.2. Analysis of Microphotographs of Cellulose

Representative microphotographs of cellulose were also obtained, where one of the biomass celluloses was shown.

**Figure 2:**
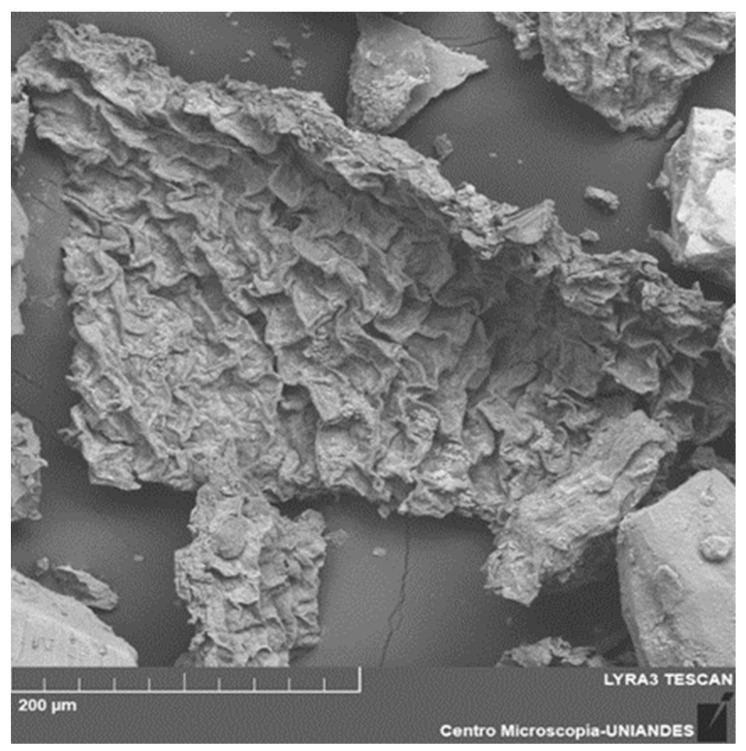
Microphotograph of cellulose of *E. crassipes*.

Figure 2 shows a characteristic image of the biomass of a cellulose sample, with a rough inhomogeneous porosity; these characteristics were also observed in [52].

The analysis of cellulose samples was carried out in physicochemical characterizations through EDS (Energy Dispersive X-ray Spectroscopy). Table 1 shows the characterization and representative percentages of this sample, where it can be seen that carbon and oxygen were the representative elements of this biomass. Different characterizations are shown based on research by [53,54,55,56].

**Figure 3:**
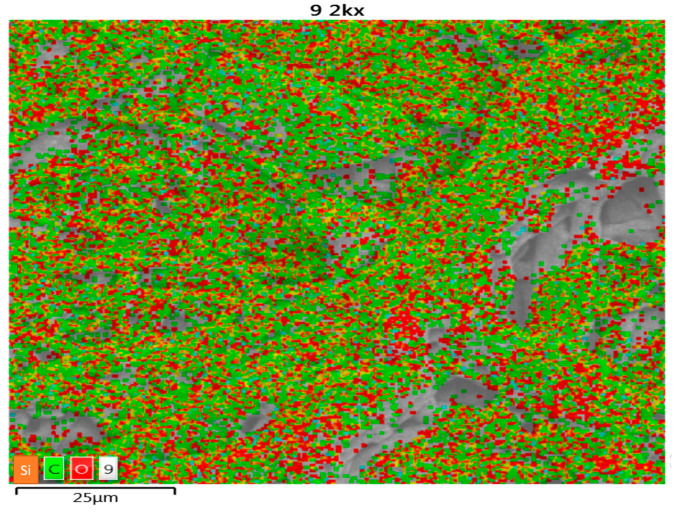
Microphotograph of cellulose of *E. crassipes* with colors.

Figure 3 shows the microphotograph of cellulose with the location of each representative element in the photomicrograph.

**Figure 4:**
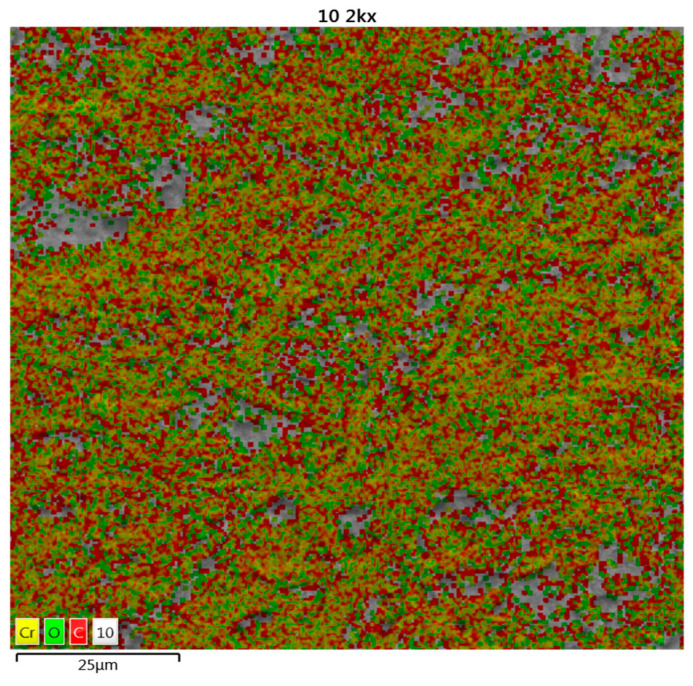
Cellulose of *E. crassipes* micrograph after treatment.

Figure 4 represents a micrograph of a general sample of cellulose, where the characteristic of its amounts of carbon and oxygen—representative elements of cellulose—and silicon, in small traces, can be observed and where cation exchange with heavy metals is possible. Table 1 shows the percentages obtained from the cellulose.

Table 2 shows the percentages of these elements, but with an arithmetic average of the different heavy metals adsorbed from the research [9,18,20,57,58,59,60,61].

Subsequently, after the adsorption treatment process of some heavy metals, the high levels of heavy metals were evident in the microphotograph, as can be seen in Figure 4.

Figure 4 shows cellulose after a treatment process. Different colored dots representing the elements in the samples can be seen, with yellow dots showing a heavy metal, red dots representing carbon, and green dots being oxygen.

### 2.3. Adsorption Mechanism

**Figure 5:**
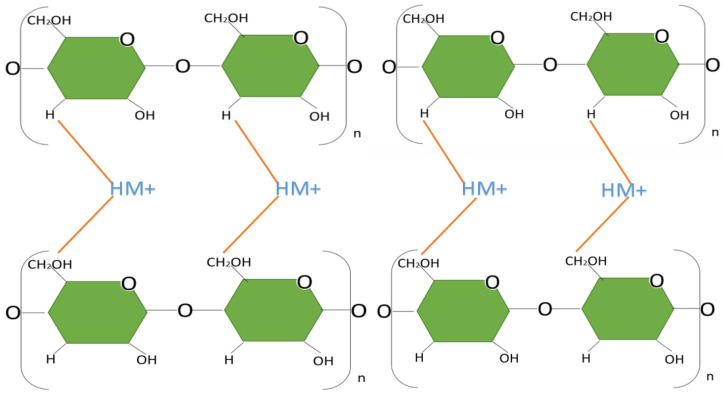
Representations of adsorptions.

In cellulose, there are hydroxyl (OH) groups where the positive ions of heavy metals are accommodated. Figure 5 shows a representative figure of heavy metal adsorption on cellulose based on different investigations [46,62,63]. The properties of this biomass, such as its hydrophilic or hydrophobic character, elasticity, water absorbance, ionic exchange or adsorption capacity, and thermal resistance, help to incorporate chemical agents to optimize the adsorption or elution process [64,65].

Figure 5 shows a generic representation of cellulose adsorption mechanisms towards heavy metals represented by Heavy Metal (HM^+^) for every four glucose molecules forming the branching of the cellulose structure (n); these deductions have been observed in [63,64,65,66,67,68,69,70,71]. Two molecules of the contaminant were attached due to chemisorption provided by the hydroxyl groups OH. 

The use of Fe (III) iron chloride in vegetable cellulose has been used to treat organic and inorganic contaminants. Fe (III) oxidizes the cellulose, forming iron hydroxides (FeOO), which is where the process of chromium diffusion by chemisorption takes place, performing a series of cation exchanges [63]. The structure of cellulose with iron chloride [62] can be seen to react with the chromium structure (VI).

**Figure 6:**
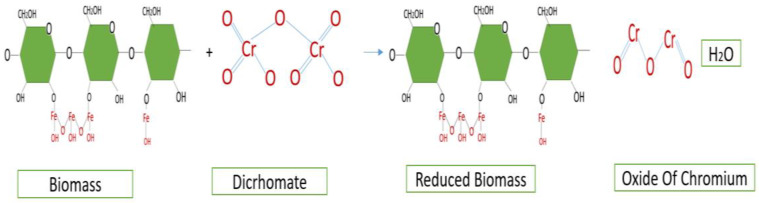
Process for reducing Cr (VI) to Cr (III).

Upon reaction with cellulose, iron chloride FeCl_3_ progressively oxidizes it, creating active sites for heavy metal adsorption [66]; chlorine reacts with the hydrogen-forming compounds of biomass (HCl). The Figure 6 show the process for reducing Cr (VI) to Cr (III).

**Figure 7:**
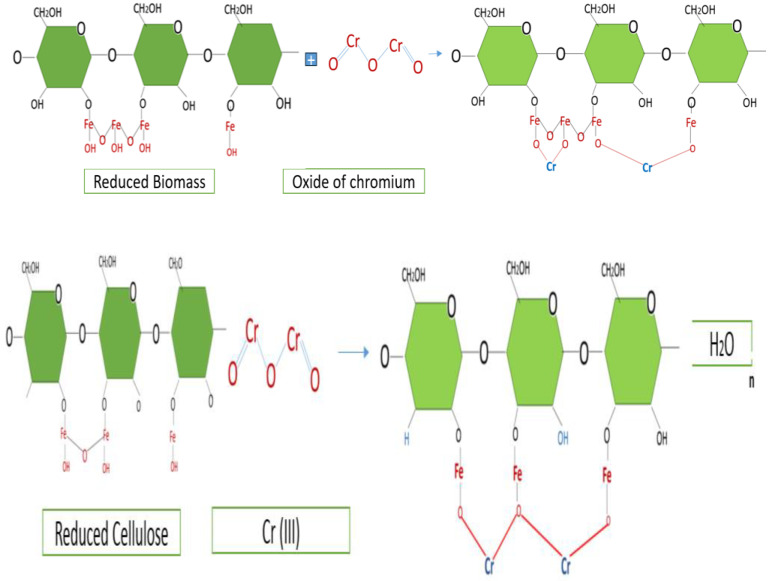
Adsorptions of Cr (VI).

Figure 7 shows the reduced biomass, in which the Cr (VI) contaminant, in the form of Cr_2_O_7_ dichromate, has a complex chemical structure. When it comes into contact with the biomass, reactions of (H^+^) in the biomass with the oxygen in the Cr (VI) structure occur, reducing it to Cr (III) [72,73,74]. Cr_2_O_2_ is chromium oxide.

## 3. Design of the Process of Treatment through the Balance of Mass and Extra-Particle and Intraparticle Diffusion

### 3.1. Mass Balance in Treatment

A desorption–elution process is involved for the reuse of biomass, see Equation (1), and this equation was designed in [18].

(1) $$q_{T}=\overset{n}{\underset{j=1}{\sum }}\left[\frac{\mathrm{QTbjCo}}{M}-\frac{\mathrm{QTbjCfj}}{M}-\frac{\varepsilon \mathrm{VCo}}{M}\right]$$

where:

Q = design flow (mL/min);
Tbj = break time of use number j (Min);
Ci = initial concentration (mg/mL);
Co = final heavy metals concentration in the treated solution (mg/mL);
V = volume of the system (mL);
Ε = porosity;
M = amount of biomass used (g);
*q_T_* = total adsorption capacity of the biomass used (mg/g).

### 3.2. Extra-Particle Diffusion

Equation (2) is the balance when the treatment process begins, and *K_f_* can be obtained through this, which is the diffusion in the external liquid film.

(2) $$Ln\;\frac{C0\left(VI\right)\;}{Cs}=\frac{kf}{l}t$$

*Kl* = Mass transfer coefficient in the liquid particle m/h;
*L*: Length;
*Cs* = Equilibrium concentration of pollutant.

Plotting this term, the natural logarithmic of initial and final concentration, with the volume and area of treated water in the experimental biotreatment process, will find the diffusion constant (*k_f_*) of in the biomass. 

### 3.3. Intraparticle Diffusion

The calculation of ma ∂q/∂t of the adsorption capacity (q) is related to the particle density (dp), and (*ks*) is the internal mass transfer coefficient and (*qs* − $\bar{q}$) [20].

(3) $$\frac{\partial q}{\partial t}=\mathrm{dpKs}\left(\mathrm{qs}-q\right)$$

where (*Cs*) is the maximum concentration of heavy metal in (mg/L) in the liquid, and (*qs*) is the concentration inside the biomass, practically its maximum capacity; the value of (*qs*) can be calculated using the following expressions, depending on the isotherm.

(4) $$\mathrm{ksdp}\left(\mathrm{qs}-q\right)=-\frac{\mathrm{Kf}}{L}\left(c-\mathrm{cs}\right)$$

The term on the left-hand side represents the rate of accumulation of the metal in the constant volume solution, which decreases because the metal is adsorbed by the cellulose. The right side represents the speed with which the metal is diffused from within the solution to the outer surface of the biomass. 

### 3.4. Modeling Process

By means of Equation (1) and with different bibliographic references, representative data were obtained to feed this equation, determining the capacities of each of these biomasses together with the new capacities, and determining the reuse power of the different solvents, as shown and summarized in Table 3.

Cellulose has excellent biochemical characteristics for developing recycling processes through chemical elutions [75,76,77,78,79,80,81,82,83,84,85]. Applying Equation (1), an increase in adsorption capacity of over 100% is evidenced in all the processes.

Untreated cellulose does not have great capacity, so it must be improved with composite materials or chemical reagents [20]. However, this deficiency contrasts with its great capacity to resist elution via chemical agents [18].

For the EDTA eluent, satisfactory results were evidenced in terms of elutions and the recycling of the cellulosic biomass of *E. crassipes* when removing Cr (VI), reaching almost 150% of its original capacity. The eluents HCl and HNO_3_ are also excellent chemical agents in the desorption process, since they achieve more than a 100% reusing of the biomass. Treatments using HCl elution represented an increase in capacities of 100%, but in all processes it acidified the cellulose, and this affected it in other adsorption processes. Elution with chemical agents is fundamental to the design of treatment systems with cellulosic biomass since, with this parameter, the removal of pollutants could be optimized [18].

Through Equation (2), the data corresponding to the investigations were linked to obtaining the extra-particle diffusion constants *K_f_*. The model establishes the input and output ratio of the pollutant as a function of the contact area, the volume of water to be treated, and the treatment time. All the models were adjusted from 500 mg/L to the 1 mg/L final quantity of the studied pollutant with a standard initial biomass of 70 g.

By obtaining *K_f_*, a treatment model of the amount of water that could be treated could be adjusted; for example, in [9], 0.20 cm/min *K_f_* was obtained with the biomass of *E. crassipes*. This system could treat 3.5 L of water. Table 4 shows a summary of the investigations.

In the references of [20,86], ideal treatment yields were obtained, adjusting 11 and 10 L, respectively. FeO gradually oxidizes this biomass, negatively charging it, allowing heavy metal ions to form chelate complexes with the oxidized sites [64]. Using a biomass such as alginate, a treatment of about 7.2 L of contaminated water could be obtained, but with a modified biomass such as *Cystoseria* + Ca and chelating cellulose, better yields could be obtained; however, the production costs of these biomasses must be established on a larger scale. With cellulose aerogel biomass and cellulose carboxymethylate giving significant results due to the high adsorption capacity of the model, these biomasses can have great value in the treatment process in the fouling industry. Cellulose xanthate consists of transformation with carbon disulfide (CS_2_), which is an alkaline biomass loaded with ions (OH^−^) that allows the easy chemisorption of metal cations [18]. The incorporation of titanium oxide (TIO_2_) increases the active sites in the biomass, such as hydroxyl groups (OH) and sites where heavy metals will lodge through cation exchange [89]. EDTA is a chemical agent that enhances the adsorption process of contaminants and is used as a chelating agent in industrial processes due to its high capacity to attract heavy metals through cation exchange [91,92,93]. Table 5 shows the intraparticle constants together with their isotherms.

Equation (3), where *K_s_* is represented, is a function of capacity, particle diameter (which must be less than 0.212 mm) [9], biomass density, and microparticle density. Each of these parameters is considered for the best fit of the constant *k_s_*.

The adsorption capacity *K_s_* was 0.0198 s for *E. crassipes* biomass, this being the biomass with the lowest yields fed by the speed of adsorption capacities, but it did not have any chemical agent, and that could be important when creating a system with this biomass. Cellulose has a ratio between the adsorbent bed and particle density, which makes it ideal in its design to create treatment systems on a larger scale, unlike other types of biomasses, for example, bacterial cellulose [97], which has a very wide adsorbent bed, which prevents better performance; the same happens with chitosan [88,89].

Biomasses fitted to the Langmuir isotherm set monolayer conditions, such as chitosan, *E. crassipes*, alginate, and biochar, where the constants were adjusted to their best rates of contaminant adsorption capacity. Therefore, this isothermal model assumes that adsorption occurs at specific homogeneous sites on the cellulose [98,99,100,101].

The Langmuir model represents the experimental data of heavy metal adsorption on cellulose better than the other adsorption models [102,103,104,105,106]. The equilibrium experimental results with the Langmuir isotherm indicate that the cellulose biomass formed a monolayer cover and that the sites on the adsorbent surface were homogeneous, together with pseudo-second order kinetic experimental results.

## 4. Design of System with the Cellulose

**Figure 8:**
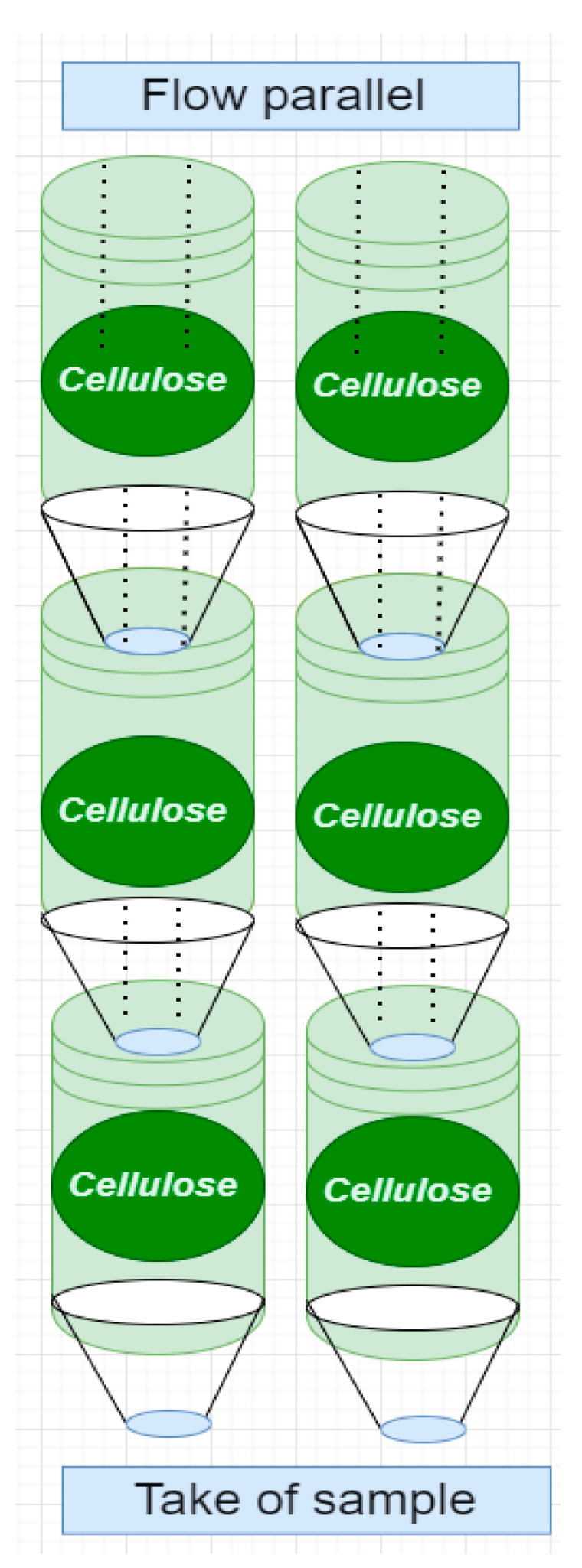
Design of the cellulose process.

Figure 8 shows a system with cellulose based on the different investigations, intending to develop a new way of treating contaminated water. The main idea is to scale up all these investigations and carry them out in order to disrupt polluting sectors; for this reason, this generic design was proposed with the idea of developing it through Equations (1)–(3), together with the elution processes.

**Figure 9:**
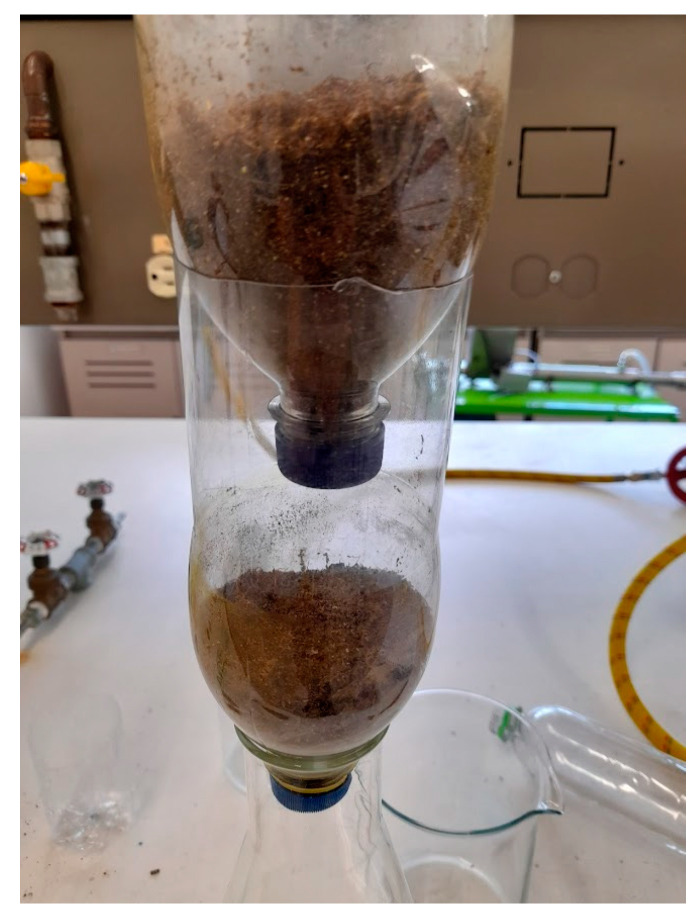
Process of treatment [9].

The treatment systems could be built with recycled PET bottles (400 and 1000 mL) (See Figure 9). Capsules or compartments were built to house the cellulose, with openings in the caps at the bottom of each capsule to allow the flow of treated water to the next capsule, as shown in Figure 9; the two processes would be used in parallel, dividing the flow into two, with three cellulose compartments each. Fixed bed column adsorption tests can be used to establish the efficiency of biomass in pollutant removal, in a similar arrangement to that used in industry [32,33,34].

The dry, shredded biomass was passed through a 60-mesh screen, allowing 0.212 mm diameter particles to pass through. The flow must be guaranteed in the upper capsule, conserving the system flow, and the system must have a manual flow control [9]. 

With the biomass of *E. crassipes* modified with iron chloride, a serial system was developed to treat water contaminated with Cr (VI) with an adsorption capacity of 18 mg/g [20]; with the same biomass but modified with cellulose xanthate, a parallel treatment system was being developed [18]. With cellulose aerogels [84], it was possible to develop a treatment system with EDTA elutions due to their high adsorption capacity of about 300 mg/g [81].

## 5. Perspectives in Cost of Implementation

Another important aspect for evaluating the process is the cost of the adsorbent materials; consequently, this section shows the estimated cost involved in obtaining them.

The characterization of the costs of the treatment systems through cellulose was elaborated on through unit costs of production of 1 kg, which were considered for drying, crushing, chemical reagents, and logistics for obtaining cellulose. The ratio with the adsorption capacity (mg/g) gives the (g HM^+^/USD) dollar spent, relating cost to adsorption capacity. Table 6 shows costs related to treatment systems.

One way to reflect the sustainability of a project is to link benefits and costs to obtain a monetary indicator [106]. Cost vs. benefit explicitly or implicitly involves cost versus total possible benefits to select the best or most cost-effective option [107]. The benefit of this project is represented through the adsorption capacity of Cr (VI) by each of the evaluated biomasses.

To establish feasibility in the development of a processing and treatment system, for example, the biomass of *E. crassipes* and its chemical modifications have excellent indicators. The best indicator is cellulose aerogels at 15 g HV^+^/USD, given the efficiency of this biomass in adsorption capacity. Cellulose clay had a value of 12.7, which is an important indicator. A suitable criterion for selecting cellulosic biomass is the geographic status; for example, in wetlands where *E. crassipes* or other plants grow, it can be decided to develop treatment systems with this biomass. On coasts, the alternatives could be marine, such as algae, but if their effluents have very large loads of heavy metals, specialized biomass such as clay–cellulose composites and cellulose aerogels could be used. Studies have revealed that cellulose waste has the potential to remove heavy metal ions in polluted water. Coupled with the adsorption capacity and costs associated with its development, this material was found to be efficient, capable of treating about 11 g of heavy metal per dollar g HM^+^/(USD), taking into account the replacements in the elutions.

## 6. Conclusions

Through the bibliographic review, it was established that the cellulose adsorption mechanism is reinforced by a microparticle–contaminant relationship, and this can be achieved through a particle diameter of less than 0.212 mm. 

An adjustment process of several parameters established in the different cellulose investigations for the removal of heavy metals has been developed, finding new adsorption capacities that have not been considered, due to studies of regeneration and reuse of cellulose adsorbents, where it was evidenced that the adsorption capacity increased considerably due to the ability of this polysaccharide to tolerate chemical reagents.

To calibrate, adjust and model a wastewater treatment system contaminated with heavy metals in continuous processes, it is ideal for due regulatory compliance to use the extra-particle diffusion model, since this function of concentrations required initial and final pollutants, together with the contact area of the biomass and the volume to be treated, in which several processes were suggested and the different extra-particle diffusion constants were calibrated.

Using the adsorption capacity and mass balance modeling equations, it was determined that cellulose, with a more compact density, has ideal diffusion constants in the design of industrial wastewater treatment systems contaminated with heavy metals in flow systems, whether continuous, in series or in parallel.

Through a cost–benefit analysis, where the unit cost of different investigations used and the adsorption capacities were considered, it was determined that cellulose has highly viable indicators when developing treatment systems.

Future studies should be carried out on a larger scale with a view to the industrial applications of this biomass, since in addition to its good adsorption capacity, it presents several advantages such as low cost. 

## Figures and Tables

**Table 1:** Physicochemical characterization of the cellulose sample.

Element	Weight	%
Carbon	53.86	60.96
Oxygen	45.62	38.76
Silicon	0.14	0.07

**Table 2:** Physicochemical characterization of cellulose with heavy metals.

Element	Weight	%
Carbon	52.2.86	56.96
Oxygen	45.62	33.76
Heavy metals	12.1	10.3

**Table 3:** Research of elutions processes.

Reference	Biomass	Contaminate Treated	Recycling	Capacity (mg/g)	Capacity (mg/g) with the Equation (1)
[20]	*E. crassipes* + Fe	Cr (VI)	EDTA	16	46
[20]	*E. crassipes*	Cr (VI)	EDTA	11	29
[75]	*Phanera vahlii*	Cr (VI)	NaOH	30	62
[76]	*A. barbadensis Miller*	Ni (II)	HCl	14	20
[77]	Green synthesized nanocrystalline chlorapatite	Cr (VI)	NaOH	20	35
[78]	Graphite	Cr (VI)	HNO_3_	20	52
[79]	Pine cone shell	Pb (II)	HCl	22	30
[18]	Xantate of cellulose	Cr (VI)	EDTA	16	51
[18]	Cellulose alkaline	Cr (VI)	EDTA	11	32
[80]	Biochar	Cd (II)	HNO_3_	15	40
[80]	Biomass	Cd (II)	HCl	11	31
[81]	Carboxymethylated Cellulose	Pb (II)	NaOH	20	48
[82]	nanofibrillated cellulose/chitosan aerogel	Cr (VI)	EDTA	50	87
[83]	Clay–cellulose biocomposite	Cd (II)	NaOH	20	115
[84]	Cellulose aerogels	Cu (II)	EDTA	40	300

**Table 4:** *K_f_* adjustment models and possible volume of water to be treated.

Reference	Biomass	Contaminate Treated	Constant *K_f_*	Volumes of Water
[9]	*E. crassipes*	Cr (VI)	0.20	3.5
[86]	*Cystoseria* + Ca + Fe	Th (IV)	0.35	4.2
[87]	Cellulose quelant	Ni (II)	0.77	8.2
[88]	Alginate	Pb (II)	0.66	7.2
[89]	*Citrus maxima peel*	Cr (VI)	0.55	5.3
[20]	*E. crassipes* + Fe	Cr (VI)	0.99	11
[18]	Xantate of cellulose	Cr (VI)	0.79	10
[90]	Sodium TiO_2_ nanofibers	Pb (II)	0.99	10
[91]	Rice bran	Cr (VI)	0.76	7.3
[92]	MXene and chitosan	Cr (VI)	0.66	5.9
[89]	Chitosan-modified magnetic carbon	Cr (VI)	0.3	3.8
[85]	Carboxymethylated Cellulose	Pb (II)	1	14
[82]	Cellulose/chitosan aerogel	Cr (VI)	0.9	11
[83]	Cellulose aerogels	Cu (II)	1.2	12

**Table 5:** Model of intraparticle diffusion and isotherm adjustments.

Reference	Biomass	Contaminate Treated	Constant *k_s_* (s)	Isotherm
[9]	*E.* *crassipes*	Cr (VI)	0.0198	Lagmuir
[86]	*Cystoseria* + Ca	Th (IV)	0.025	Temkin
[87]	Cellulose quelant	Ni (II)	0.077	Freundlinch
				Langmuir
[88]	Alginate	Pb (II)	0.033	Langmuir
[89]	Citrus maxima peel	Cr (VI)	0.22	Freundlinch
[20]	*E. crassipes* + Fe	Cr (VI)	0.045	Langmuir
[18]	Xantate of cellose	Cr (VI)	0.04	Freundlinch
[90]	Sodium titanate nanofibers	Pb (II)	0.04	Temkin
[94]	Rice bran	Cr (VI)	0.035	Langmuir
[95]	MXene and chitosan	Cr (VI)	0.02	Freunlinhchd
[96]	Chitosan-modified magnetic carbon	Cr (VI)	0.03	Langmuir
[85]	Carboxymethylate Cellulose	Pb (II)	0.08	Langmuir
[82]	Cellulose/chitosan aerogel	Cr (VI)	0.075	Langmuir
[84]	Cellulose aerogels	Cu (II)	0.08	Langmuir

**Table 6:** Costs related to treatment systems.

Reference	Biomass	Recycling	Cost (USD) 1 kg Material	Capacity (mg/g)	g HM^+^/(USD)
[20]	*E. crassipes* + Fe	EDTA	4	46	11.5
[20]	*E. crassipes*	EDTA	3	29	9.6
[75]	*Phanera vahlii*	NaOH	6	62	10
[79]	Pine cone shell	HCl	4	30	7.5
[18]	Xantate of cellulose	EDTA	6	51	9
[18]	Cellulose alkaline	EDTA	4	32	8
[80]	Biochar	HNO_3_	8	40	5
[82]	Cellulose/chitosan aerogel	EDTA	10	87	8.7
[83]	Clay-cellulose biocomposite	NaOH	20	115	12.7
[84]	Cellulose aerogels	EDTA	25	300	15

## Data Availability

The datasets used and/or analyzed during the current study are available from the corresponding author upon reasonable request.

## References

[1] Kumar V., Parihar R.D., Sharma A., Bakshi P., Sidhu G.P.S., Bali A.S., Karaouzas I., Bhardwaj R., Thukral A.K., Gyasi-Agyei Y. (2019). Global evaluation of heavy metal content in surface water bodies: A meta-analysis using heavy metal pollution indices and multivariate statistical analyses. Chemosphere.

[2] Briffa J., Sinagra E., Blundell R. (2020). Heavy metal pollution in the environment and their toxicological effects on humans. Heliyon.

[3] Rahman M.A., Paul M., Bhoumik N., Hassan M., Alam M.K., Aktar Z. (2020). Heavy metal pollution assessment in the groundwater of the Meghna Ghat industrial area, Bangladesh, by using water pollution indices approach. Appl. Water Sci..

[4] Khadija D., Hicham A., Rida A., Hicham E., Nordine N., Najlaa F. (2021). Surface water quality assessment in the semi-arid area by a combination of heavy metal pollution indices and statistical approaches for sustainable management. Environ. Chall..

[5] Mahamood M., Khan F.R., Zahir F., Javed M., Alhewairini S.S. (2023). Bagarius bagarius, and *Eichhornia crassipes* are suitable bioindicators of heavy metal pollution, toxicity, and risk assessment. Sci. Rep..

[6] Khan M., Javed M., Rehman M.T., Urooj M., Ahmad M.I. (2020). Heavy metal pollution and risk assessment by the battery of toxicity tests. Sci. Rep..

[7] Kapahi M., Sachdeva S. (2019). Bioremediation options for heavy metal pollution. J. Health Pollut..

[8] Karaouzas I., Kapetanaki N., Mentzafou A., Kanellopoulos T.D., Skoulikidis N. (2020). Heavy metal contamination status in Greek surface waters: A review with application and evaluation of pollution indices. Chemosphere.

[9] Sayago UF C. (2021). Design and development of a biotreatment of *E. crassipes* for the decontamination of water with Chromium (VI). Sci. Rep..

[10] Carreño-Sayago U.F. (2021). Development of microspheres using water hyacinth (*Eichhornia crassipes*) for treatment of contaminated water with Cr (VI). Environ. Dev. Sustain..

[11] Patel H. (2020). Batch and continuous fixed bed adsorption of heavy metals removal using activated charcoal from neem (*Azadirachta indica*) leaf powder. Sci. Rep..

[12] Akindele E.O., Omisakin O.D., Oni O.A., Aliu O.O., Omoniyi G.E., Akinpelu O.T. (2020). Heavy metal toxicity in the water column and benthic sediments of a degraded tropical stream. Ecotoxicol. Environ. Saf..

[13] Zia Z., Hartland A., Mucalo M.R. (2020). Use of low-cost biopolymers and biopolymeric composite systems for heavy metal removal from water. Int. J. Environ. Sci. Technol..

[14] Ma X., Zhao S., Tian Z., Duan G., Pan H., Yue Y., Li S., Jian S., Yang W., Liu K. (2022). MOFs meet wood: Reusable magnetic hydrophilic composites toward efficient water treatment with super-high dye adsorption capacity at high dye concentration. Chem. Eng. J..

[15] Mohammed Y., Song K., Li L. (2020). Fixed bed column and artificial neural network model to predict heavy metals adsorption dynamic on surfactant decorated graphene. Colloids Surf. A Physicochem. Eng. Asp..

[16] Danish M., Ansari K.B., Aftab R.A., Danish M., Zaidi S., Trinh Q.T. (2023). gPROMS-driven modeling and simulation of fixed bed adsorption of heavy metals on a biosorbent: Benchmarking and case study. Environ. Sci. Pollut. Res..

[17] Fallah N., Taghizadeh M. (2020). Continuous fixed-bed adsorption of Mo (VI) from aqueous solutions by Mo (VI)-IIP: Breakthrough curves analysis and mathematical modeling. J. Environ. Chem. Eng..

[18] Sayago UF C., Ballesteros Ballesteros V. (2023). Development of a treatment for water contaminated with Cr (VI) using cellulose xanthogenate from *E. crassipes* on a pilot scale. Sci. Rep..

[19] Chen Y., Li S., Li X., Mei C., Zheng J., E S., Duan G., Liu K., Jiang S. (2021). Liquid transport and real-time dye purification via lotus petiole-inspired long-range-ordered anisotropic cellulose nanofibril aerogels. ACS Nano.

[20] Carreño Sayago U.F., Piñeros Castro Y., Conde Rivera L.R. (2022). Design of a Fixed-Bed Column with Vegetal Biomass and Its Recycling for Cr (VI) Treatment. Recycling.

[21] Carreño Sayago U.F. (2021). Design, scaling, and development of biofilters with *E. crassipes* for treatment of water contaminated with Cr (VI). Water.

[22] Shi C., Wang X., Zhou S., Zuo X., Wang C. (2022). Mechanism, application, influencing factors and environmental benefit assessment of steel slag in removing pollutants from water: A review. J. Water Process Eng..

[23] León G., Gómez E., Miguel B., Hidalgo A.M., Gómez M., Murcia M.D., Guzmán M.A. (2022). Feasibility of adsorption kinetic models to study carrier-mediated transport of heavy metal ions in emulsion liquid membranes. Membranes.

[24] Amari A., Alawameleh H.S.K., Isam M., Maktoof M.A.J., Osman H., Panneerselvam B., Thomas M. (2023). Thermodynamic Investigation and Study of Kinetics and Mass Transfer Mechanisms of Oily Wastewater Adsorption on UIO-66–MnFe_2_O_4_ as a Metal–Organic Framework (MOF). Sustainability.

[25] Abdulrasool M.M., Ruaa K.M., Mays A.D., ALsailawi H.A., Mudhafar M., Bashi A.M. (2021). Regeneration of Chitosan-Based Adsorbents Used in Heavy Metal Adsorption. J. Life Sci..

[26] Sohrabi N., Mohammadi R., Ghassemzadeh H.R., Heris S.S.S. (2021). Design and synthesis of a new magnetic molecularly imprinted polymer nanocomposite for specific adsorption and separation of diazinon insecticides from aqueous media. Microchem. J..

[27] Jiang Q., He Y., Wu Y., Dian B., Zhang J., Li T., Jiang M. (2022). Solidification/stabilization of soil heavy metals by alkaline industrial wastes: A critical review. Environ. Pollut..

[28] Xie X., Zhang L., Luo X., Su T., Zhang Y., Qin Z., Ji H. (2022). PEI modified magnetic porous cassava residue microspheres for adsorbing Cd (II) from aqueous solution. Eur. Polym. J..

[29] Zhao B., Jiang H., Lin Z., Xu S., Xie J., Zhang A. (2019). Preparation of acrylamide/acrylic acid cellulose hydrogels for the adsorption of heavy metal ions. Carbohydr. Polym..

[30] Danish M., Ansari K.B., Danish M., Khatoon A., Rao R.A.K., Zaidi S., Aftab R.A. (2022). A comprehensive investigation of external mass transfer and intraparticle diffusion for batch and continuous adsorption of heavy metals using pore volume and surface diffusion model. Sep. Purif. Technol..

[31] Zou W., Feng X., Wang R., Wei W., Luo S., Zheng R., Yang D., Mi H., Chen H. (2021). High-efficiency core-shell magnetic heavy-metal absorbents derived from spent-LiFePO_4_ Battery. J. Hazard. Mater..

[32] Si Y., Li J., Cui B., Tang D., Yang L., Murugadoss V., Maganti S., Huang M., Guo Z. (2022). Janus phenol–formaldehyde resin and periodic mesoporous organic silica nanoadsorbent for the removal of heavy metal ions and organic dyes from polluted water. Adv. Compos. Hybrid Mater..

[33] Yu F., Li Y., Huang G., Yang C., Chen C., Zhou T., Zhao Y., Ma J. (2020). Adsorption behavior of the antibiotic levofloxacin on microplastics in the presence of different heavy metals in an aqueous solution. Chemosphere.

[34] Mittal J., Ahmad R., Mariyam A., Gupta V.K., Mittal A. (2021). Expeditious and enhanced sequestration of heavy metal ions from aqueous environment by papaya peel carbon: A green and low-cost adsorbent. Desalin. Water Treat.

[35] Shi T., Ma J., Wu F., Ju T., Gong Y., Zhang Y., Wu X., Hou H., Zhao L., Shi H. (2019). Mass balance-based inventory of heavy metals inputs to and outputs from agricultural soils in Zhejiang Province, China. Sci. Total Environ..

[36] Whitehead P.G., Bussi G., Peters R., Hossain M.A., Softley L., Shawal S., Jin L., Rampley C.P.N., Holdship P., Hope R. (2019). Modelling heavy metals in the Buriganga River System, Dhaka, Bangladesh: Impacts of tannery pollution control. Sci. Total Environ..

[37] Luo H., Wang Q., Guan Q., Ma Y., Ni F., Yang E., Zhang J. (2022). Heavy metal pollution levels, source apportionment and risk assessment in dust storms in key cities in Northwest China. J. Hazard. Mater..

[38] Hong N., Guan Y., Yang B., Zhong J., Zhu P., Ok Y.S., Hou D., Tsang D.C.W., Guan Y., Liu A. (2020). Quantitative source tracking of heavy metals contained in urban road deposited sediments. J. Hazard. Mater..

[39] Sayago UF C., Castro Y.P., Rivera LR C., Mariaca A.G. (2020). Estimation of equilibrium times and maximum capacity of adsorption of heavy metals by *E. crassipes*. Environ. Monit. Assess..

[40] Carreño Sayago U.F. (2020). “Buchón De Agua” (Eichhornia crassipes): Impulsor De La Fitorremediación.

[41] Jian S., Chen Y., Shi F., Liu Y., Jiang W., Hu J., Han X., Jiang S., Yang W. (2022). Template-Free Synthesis of Magnetic La-Mn-Fe Tri-Metal Oxide Nanofibers for Efficient Fluoride Remediation: Kinetics, Isotherms, Thermodynamics and Reusability. Polymers.

[42] Jin X., Xiang Z., Liu Q., Chen Y., Lu F. (2017). Polyethyleneimine-bacterial cellulose bioadsorbent for effective removal of copper and lead ions from aqueous solution. Bioresour. Technol..

[43] Stoica-Guzun A., Stroescu M., Jinga S.I., Mihalache N., Botez A., Matei C., Berger D., Damian C.M., Ionita V. (2016). Box-Behnken experimental design for chromium (VI) ions removal by bacterial cellulose-magnetite composites. Int. J. Biol. Macromol..

[44] Mohammed A.B., Omran A.R., Baiee M.A., Salman J.M. (2018). Biosorption of Safranin-O from Aqueous Solution by Nile Rose Plant (*Eichhornia crassipes*). Baghdad Sci. J..

[45] Wang C., Wang H., Gu G. (2018). Ultrasound-assisted xanthation of cellulose from lignocellulosic biomass optimized by response surface methodology for Pb (II) sorption. Carbohydr. Polym..

[46] Wang J., Lu X., Ng P.F., Lee K.I., Fei B., Xin J.H., Wu J.Y. (2015). Polyethylenimine coated bacterial cellulose nanofiber membrane and application as adsorbent and catalyst. J. Colloid Interface Sci..

[47] El-Naggar M.E., Radwan E.K., El-Wakeel S.T., Kafafy H., Gad-Allah T.A., El-Kalliny A.S., Shaheen T.I. (2018). Synthesis, characterization and adsorption properties of microcrystalline cellulose based nanogel for dyes and heavy metals removal. Int. J. Biol. Macromol..

[48] Jin L., Bai R. (2002). Mechanisms of lead adsorption on chitosan/PVA hydrogel beads. Langmuir.

[49] Sun S., Wang A. (2006). Adsorption kinetics of Cu (II) ions using N,O-carboxymethyl-chitosan. J. Hazard. Mater..

[50] Yuwei C., Jianlong W. (2011). Preparation and characterization of magnetic chitosan nanoparticles and its application for Cu (II) removal. Chem. Eng. J..

[51] Krishnamachari P., Hashaikeh R., Tiner M. (2011). Modified cellulose morphologies and its composites; SEM and TEM analysis. Micron.

[52] Zhou Y.T., Nie H.L., Branford-White C., He Z.Y., Zhu L.M. (2009). Removal of Cu^2+^ from aqueous solution by chitosan-coated magnetic nanoparticles modified with α-ketoglutaric acid. J. Colloid Interface Sci..

[53] Taka A.L., Klink M.J., Mbianda X.Y., Naidoo E.B. (2020). Chitosan nanocomposites for water treatment by fixed-bed continuous flow column adsorption: A review. Carbohydr. Polym..

[54] Yang X., Liu Z., Jiang Y., Li F., Xue B., Dong Z., Ding M., Chen R., Yang Q., An T. (2020). Micro-structure, surface properties and adsorption capacity of ball-milled cellulosic biomass derived biochar based mineral composites synthesized via carbon-bed pyrolysis. Appl. Clay Sci..

[55] Yu X., Tong S., Ge M., Wu L., Zuo J., Cao C., Song W. (2013). Adsorption of heavy metal ions from aqueous solution by carboxylated cellulose nanocrystals. J. Environ. Sci..

[56] Zhang N., Zang G.L., Shi C., Yu H.Q., Sheng G.P. (2016). A novel adsorbent TEMPO-mediated oxidized cellulose nanofibrils modified with PEI: Preparation, characterization, and application for Cu (II) removal. J. Hazard. Mater..

[57] Jiang H., Yang Y., Lin Z., Zhao B., Wang J., Xie J., Zhang A. (2020). Preparation of a novel bio-adsorbent of sodium alginate grafted polyacrylamide/graphene oxide hydrogel for the adsorption of heavy metal ion. Sci. Total Environ..

[58] Li H., Wang Y., Ye M., Zhang X., Zhang H., Wang G., Zhang Y. (2021). Hierarchically porous poly (amidoxime)/bacterial cellulose composite aerogel for highly efficient scavenging of heavy metals. J. Colloid Interface Sci..

[59] Tang C., Brodie P., Li Y., Grishkewich N.J., Brunsting M., Tam K.C. (2020). Shape recoverable and mechanically robust cellulose aerogel beads for efficient removal of copper ions. Chem. Eng. J..

[60] Tang P., Sun Q., Zhao L., Tang Y., Liu Y., Pu H., Gan N., Liu Y., Li H. (2019). A simple and green method to construct cyclodextrin polymer for the effective and simultaneous estrogen pollutant and metal removal. Chem. Eng. J..

[61] Sun Y., Yin W.M., Wang Y., Zhao N.D., Wang X.Y., Zhang J.G., Guo Y.R., Li S., Pan Q.J. (2022). Fabrication of ultra-thin MgAl layered double oxide by cellulose templating and its immobilization effect toward heavy metal ions: Cation-exchange and deposition mechanism. Chem. Eng. J..

[62] Abere D.V., Ojo S.A., Paredes-Epinosa M.B., Hakami A. (2022). Derivation of composites of chitosan-nanoparticles from crustaceans source for nanomedicine: A mini review. Biomed. Eng. Adv..

[63] Kang X., Cong Z., Pin X., Du Z., Cai Z. (2022). Copper ion-imprinted bacterial cellulose for selectively removing heavy metal ions from aqueous solution. Cellulose.

[64] Hokkanen S., Repo E., Lou S., Sillanpää M. (2011). Removal of arsenic (V) by magnetic nanoparticle activated microfibrillated cellulose. Chem. Eng. J..

[65] Chen A., Zeng G., Chen G., Hu X., Yan M., Guan S., Shang C., Lu L., Zou Z., Xie G. (2012). Novel thiourea-modified magnetic ion-imprinted chitosan/TiO_2_ composite for simultaneous removal of cadmium and 2,4-dichlorophenol. Chem. Eng. J..

[66] Lin S., Yang H., Na Z., Lin K. (2018). A novel biodegradable arsenic adsorbent by immobilization of iron oxyhydroxide (FeOOH) on the root powder of long-root *Eichhornia crassipes*. Chemosphere.

[67] Huang X., Zhan X., Wen C., Xu F., Luo L. (2018). Amino-functionalized magnetic bacterial cellulose/activated carbon composite for Pb^2+^ and methyl orange sorption from aqueous solution. J. Mater. Sci. Technol..

[68] Okieimen F.E., Sogbaike C.E., Ebhoaye J.E. (2005). Removal of cadmium and copper ions from aqueous solution with cellulose graft copolymers. Sep. Purif. Technol..

[69] Bringas A., Bringas E., Ibañez R., San-Román M.F. (2023). Fixed-bed columns mathematical modeling for selective nickel and copper recovery from industrial spent acids by chelating resins. Sep. Purif. Technol..

[70] Sounthararajah D.P., Loganathan P., Kandasamy J., Vigneswaran S. (2015). Adsorptive removal of heavy metals from water using sodium titanate nanofibres loaded onto GAC in fixed-bed columns. J. Hazard. Mater..

[71] Bhatti H.N., Mahmood Z., Kausar A., Yakout S.M., Shair O.H., Iqbal M. (2020). Biocomposites of polypyrrole, polyaniline and sodium alginate with cellulosic biomass: Adsorption-desorption, kinetics and thermodynamic studies for the removal of 2,4-dichlorophenol. Int. J. Biol. Macromol..

[72] Yang W., Wang Y., Wang Q., Wu J., Duan G., Xu W., Jian S. (2021). Magnetically separable and recyclable Fe_3_O_4_@ PDA covalent grafted by l-cysteine core-shell nanoparticles toward efficient removal of Pb^2+^. Vacuum.

[73] Wang J., Sun Y., Zhao X., Chen L., Peng S., Ma C., Duan G., Liu Z., Wang H., Yuan Y. (2022). A poly (amidoxime)-modified MOF macroporous membrane for high-efficient uranium extraction from seawater. e-Polymers.

[74] Chen Q., Zheng J., Wen L., Yang C., Zhang L. (2019). A multi-functional-group modified cellulose for enhanced heavy metal cadmium adsorption: Performance and quantum chemical mechanism. Chemosphere.

[75] Ajmani A., Shahnaz T., Subbiah S., Narayanasamy S. (2019). Hexavalent chromium adsorption on virgin, biochar, and chemically modifed carbons prepared from Phanera vahlii fruit biomass: Equilibrium, kinetics, and thermodynamics approach. Environ. Sci. Pollut. Res..

[76] Gupta S., Jain A.K. (2021). Biosorption of Ni (II) from aqueous solutions and real industrial wastewater using modifed *A. barbadensis* Miller leaves residue powder in a lab scale continuous fxed bed column. Clean. Eng. Technol..

[77] Han X., Zhang Y., Zheng C., Yu X., Li S., Wei W. (2021). Enhanced Cr (VI) removal from water using a green synthesized nanocrystalline chlorapatite: Physicochemical interpretations and fxed-bed column mathematical model study. Chemosphere.

[78] Ghasemabadi S.M., Baghdadi M., Safari E., Ghazban F. (2018). Investigation of continuous adsorption of Pb (II), As (III), Cd (II), and Cr (VI) using a mixture of magnetic graphite oxide and sand as a medium in a fxed-bed column. J. Environ. Chem. Eng..

[79] Martín-Lara M.A., Blázquez G., Calero M., Almendros A.I., Ronda A. (2016). Binary biosorption of copper and lead onto pine cone shell in batch reactors and in fxed bed columns. Int. J. Miner. Process..

[80] Abdolali A., Ngo H.H., Guo W., Zhou J.L., Zhang J., Liang S., Chang S.W., Nguyen D.D., Liu Y. (2017). Application of a breakthrough biosorbent for removing heavy metals from synthetic and real wastewaters in a lab-scale continuous fixed-bed column. Bioresour. Technol..

[81] Xiang T., Zhang Z., Liu H., Yin Z., Li L., Liu X. (2013). Characterization of cellulose-based electrospun nanofiber membrane and its adsorptive behaviours using Cu (II), Cd (II), Pb (II) as models. Sci. China Chem..

[82] Wang Q., Zuo W., Tian Y., Kong L., Cai G., Zhang H., Li L., Zhang J. (2023). An ultralight and flexible nanofibrillated cellulose/chitosan aerogel for efficient chromium removal: Adsorption-reduction process and mechanism. Chemosphere.

[83] Abu-Danso E., Peräniemi S., Leiviskä T., Kim T., Tripathi K.M., Bhatnagar A. (2020). Synthesis of clay-cellulose biocomposite for the removal of toxic metal ions from aqueous medium. J. Hazard. Mater..

[84] Kardam A., Raj K.R., Srivastava S., Srivastava M.M. (2014). Nanocellulose fibers for biosorption of cadmium, nickel, and lead ions from aqueous solution. Clean Technol. Environ. Policy.

[85] Islam M.S., Rahaman M.S., Barbeau B. (2023). Single and Multi Component Removal of Pb+2, Zn+2, Cu+2, and As+3 Ions from Aqueous Solution Using Kraft Pulp-Based Carboxymethylated Cellulose by Fixed-Bed Column Adsorption Process. SSRN Electron. J..

[86] Amiri M., Keshtkar A.R., Moosavian M.A. (2022). Th (IV) biosorption from a three-component feed solution by Ca-pretreated Cystoseria indica alga in a fixed-bed column: Experimental tests and modeling. J. Environ. Chem. Eng..

[87] Jiang X., An Q.D., Xiao Z.Y., Zhai S.R., Shi Z. (2019). Versatile core/shell-like alginate@ polyethylenimine composites for efficient removal of multiple heavy metal ions (Pb^2+^, Cu^2+^, CrO_4_^2−^): Batch and fixed-bed studies. Mater. Res. Bull..

[88] Jain M., Garg V.K., Kadirvelu K. (2013). Cadmium (II) sorption and desorption in a fixed bed column using sunflower waste carbon calcium–alginate beads. Bioresour. Technol..

[89] Chao H.P., Chang C.C., Nieva A. (2014). Biosorption of heavy metals on Citrus maxima peel, passion fruit shell, and sugarcane bagasse in a fixed-bed column. J. Ind. Eng. Chem..

[90] Peng J., Yuan H., Ren T., Liu Z., Qiao J., Ma Q., Wu Y. (2022). Fluorescent nanocellulose-based hydrogel incorporating titanate nanofibers for sorption and detection of Cr (VI). Int. J. Biol. Macromol..

[91] Chatterjee A., Abraham J. (2019). Desorption of heavy metals from metal loaded sorbents and e-wastes: A review. Biotechnol. Lett..

[92] Guo Z., Hou H., Zhou J., Wu X., Li Y., Hu L. (2023). Fabrication of novel 3D PEI-functionalized ZIF-8@ alginate aerogel composites for efficient elimination of Pb (II) and Cd (II) from aqueous solution. J. Environ. Chem. Eng..

[93] Júnior W.N., Silva MG C., Vieira M.G.A. (2020). Competitive fixed-bed biosorption of Ag (I) and Cu (II) ions on *Sargassum filipendula* seaweed waste. J. Water Process Eng..

[94] Sarkar S., Bar N., Das S.K. (2021). Cr (VI) and Cu (II) removal from aqueous solution in fixed bed column using rice bran; experimental, statistical and GA modelling. J. Indian Chem. Soc..

[95] Asif U.A., Mahmood K., Naqvi S.R., Mehran M.T., Noor T. (2023). Development of high-capacity surface-engineered MXene composite for heavy metal Cr (VI) removal from industrial wastewater. Chemosphere.

[96] Aslam MM A., Den W., Kuo H.W. (2021). Removal of hexavalent chromium by encapsulated chitosan-modified magnetic carbon nanotubes: Fixed-bed column study and modelling. J. Water Process Eng..

[97] Sayago UF C., Castro Y.P. (2022). Development of a composite material between bacterial cellulose and *E. crassipes*, for the treatment of water contaminated by chromium (VI). Int. J. Environ. Sci. Technol..

[98] Ammar N.S., Elhaes H., Ibrahim H.S., Ibrahim M.A. (2014). A novel structure for removal of pollutants from wastewater. Spectrochim. Acta Part A Mol. Biomol. Spectrosc..

[99] Deng L., Geng M., Zhu D., Zhou W., Langdon A., Wu H., Yu Y., Zhu Z., Wang Y. (2012). Effect of chemical and biological degumming on the adsorption of heavy metal by cellulose xanthogenates prepared from *Eichhornia crassipes*. Bioresour. Technol..

[100] El-Zawahry M.M., Abdelghaffar F., Abdelghaffar R.A., Hassabo A.G. (2016). Equilibrium and kinetic models on the adsorption of reactive black 5 from aqueous solution using *Eichhornia crassipes*/chitosan composite. Carbohydr. Polym..

[101] Jian S., Cheng Y., Ma X., Guo H., Hu J., Zhang K., Jiang S., Yang W., Duan G. (2022). Excellent fluoride removal performance by electrospun La–Mn bimetal oxide nanofibers. New J. Chem..

[102] Feng W., Xiao K., Zhou W., Zhu D., Zhou Y., Yuan Y., Xiao N., Wan X., Hua Y., Zhao J. (2017). Analysis of utilization technologies for *Eichhornia crassipes* biomass harvested after restoration of wastewater. Bioresour. Technol..

[103] Lin S., Wang G., Na Z., Lu D., Liu Z. (2012). Long-root *Eichhornia crassipes* as a biodegradable adsorbent for aqueous as (III) and as (V). Chem. Eng. J..

[104] Liu L., Hu S., Shen G., Farooq U., Zhang W., Lin S., Lin K. (2018). Adsorption dynamics and mechanism of aqueous sulfachloropyridazine and analogues using the root powder of recyclable long-root *Eichhornia crassipes*. Chemosphere.

[105] Man Q., An Y., Shen H., Wei C., Zhang X., Wang Z., Feng J. (2023). MXenes and Their Derivatives for Advanced Solid-State Energy Storage Devices. Adv. Funct. Mater..

[106] Logar I., Brouwer R., Paillex A. (2019). Do the societal benefits of river restoration outweigh their costs? A cost-benefit analysis. J. Environ. Manag..

[107] Brown M., Snelling E., De Alba M., Ebrahimi G., Forster B.B. (2023). Quantitative Assessment of Computed Tomography Energy Use and Cost Savings Through Overnight and Weekend Power Down in a Radiology Department. Can. Assoc. Radiol. J..

